# Prevalence of Larval Breeding Sites and Seasonal Variations of *Aedes* *aegypti* Mosquitoes (Diptera: Culicidae) in Makkah Al-Mokarramah, Saudi Arabia

**DOI:** 10.3390/ijerph18147368

**Published:** 2021-07-09

**Authors:** Elfadol Obeid Mohamed Ali, Ahmed Omer Babalghith, Adil Omer Saeed Bahathig, Fawzia Hassan Suleiman Toulah, Turki Ghazi Bafaraj, Sameer Mohammed Yousef Al-Mahmoudi, Abdullah Mousa Fawaz Alhazmi, Mohamed E. Abdel-Latif

**Affiliations:** 1 Faculty of Public Health and Health Informatics, Umm Al-Qura University, Makkah 24230, Saudi Arabia; alhazmi15@hotmail.com; 2 Faculty of Medicine, Umm Al-Qura University, Makkah 24230, Saudi Arabia; aobabalghith@uqu.edu.sa (A.O.B.); aobahathiq@uqu.edu.sa (A.O.S.B.); 3 Department of Zoology Faculty of Science, AL Faisaliah Campus, King Abdulaziz University, Jeddah 21577, Saudi Arabia; ftoulah@kau.edu.sa; 4 Vectors Control Department, Ministry of Health, Makkah 24230, Saudi Arabia; dr.turkibafaraj@me.com (T.G.B.); abojana2120@gmail.com (S.M.Y.A.-M.); 5 The Medical School, College of Health and Medicine, Australian National University, Canberra, ACT 2601, Australia; Abdel-Latif.Mohamed@act.gov.au; 6 Department of Public Health, La Trobe University, Health Sciences Building 2, Melbourne, VIC 3083, Australia

**Keywords:** *Aedes aegypti* mosquitoes, larval ecology, Makkah, dengue fever

## Abstract

Since 1994, dengue fever (DF) transmission rates have increased significantly in Saudi Arabia (KSA). Climatic, geographic, and demographic conditions make KSA especially suitable for DF’s spread. Still, there are insufficient strategies for controlling the *Aedes* species that transmit DF virus (DENV). To develop effective management strategies, it is necessary to identify *Aedes* species and the ecological habitat of larvae in Makkah Al-Mokarramah, KSA. We conducted a longitudinal survey of *Aedes* mosquitoes in 14 localities from January 2015 to December 2015. World Health Organization (WHO) inspection kits for larvae were used to detect and sample larvae, along with pictorial keys. A total of 42,981 potential *Aedes* larval breeding sites were surveyed. A total of 5403 (12.6%) sites had at least one water source positive for *Aedes aegypti* (Linnaeus) mosquitoes. Among the total of 15,133 water sources surveyed within the sampled sites, 1815 (12.0%) were positive for *Aedes aegypti*. *Aedes aegypti* was the only *Aedes* species identified in the course of the survey. The presence of such a large immature population may indicate an imminent outbreak of DF in the near future unless proper implementation of control and elimination of *Aedes* *aegypti* are undertaken. Additionally, the adaptation of *Aedes* *aegypti* to the arid climate of Makkah needs further investigation.

## 1. Introduction

Vector-borne diseases, particularly malaria and dengue fever, are responsible for over 700,000 deaths each year and represent more than 17% of all infectious diseases worldwide [1]. Their huge impact makes them the most significant contemporary public health risk. Dengue fever (DF) is an incredibly notorious example of vector-borne disease. DF is the most common vector-borne disease worldwide and, thus, also the most important [2]. The WHO estimates that 40% of the ‘world’s population lives in DF high-risk areas. In the 30 countries most impacted by DF, approximately 390 million reported cases and 20,000 deaths from DF are reported annually. Globally, more than 2.5 billion people are threatened by DF, and the disease is a substantial burden on affected populations [3].

Dengue virus (DENV), the causative agent of DF, is primarily transmitted by the bite of the infected female *Aedes aegypti* (Linnaeus) and, to a lesser extent, *Aedes albopictus* mosquitoes [4]. DF is endemic in the regions where these mosquitoes are common: Southeast Asia, the Western Pacific, the Americas, the Eastern Mediterranean, and Africa [4].

*Aedes aegypti* (Skuse) is the principal vector for DF and its more severe manifestation, dengue hemorrhagic fever, in almost all countries [5]. It is found in urban habitats and breeds mostly in artificial containers. Unlike other mosquitoes, *Aedes aegypti* feeds during the day; its peak biting periods are early in the morning and before dusk. Females of the species bite multiple people during each feeding period [6].

The Kingdom of Saudi Arabia (KSA) is home to many of the conditions required for DF to spread. These include increasing population growth, a recent increase in the movement of individuals, water scarcity, water storage patterns, the presence of the virus, the availability of a susceptible population, and the existence of competent vectors [7]. The presence of these conditions in the KSA has contributed to a continuous increase in reported cases of DF. Furthermore, the KSA is host to a highly mobile and transient population of expatriates, workers, and pilgrims undergoing the Umrah and the Hajj. As infected travelers return from endemic areas or travel between regions of the KSA with established populations of *Aedes aegypti* or *Aedes albopictus*, local viral transmission can easily be introduced [8,9].

In the KSA, DF virus isolation was first achieved during an outbreak in 1994 in Jeddah, where 289 confirmed cases were recorded. Since then, there have continued to be sporadic DF cases in Jeddah [10,11]. There have been DF reports in Makkah since 2004, following the first outbreak in the region [12]. By 2009, the rate of DF transmission and infection in the KSA was increasing significantly; the Saudi Ministry of Health reported 3350 cases and estimated the fatality rate to be 4.6 per thousand people [13]. An association has been identified between the incidence of the disease and the presence of construction workers [14].

In the absence of an effective drug or vaccine, the potential for management of DF and its spread is limited. The only available strategies are case management of infected individuals to prevent death and personal protective measures to reduce the rate of transmission [15]. Widespread control of mosquito populations is difficult due to the complex behavior of adult mosquitoes, which makes it most practical to intervene when they are still in their aquatic, immature stages [16]. To combat the spread of DF more effectively, a more comprehensive understanding of the behavior and activity of vectors such as *Aedes aegypti* is required.

With these challenges in mind, this study aimed to (1) identify the species of *Aedes* mosquito present in Makkah Al-Mokarrama, Saudi Arabia; (2) identify the ecological habitat of larvae in this region; and (3) evaluate the spread of *Aedes aegypti* in Makkah Al-Mokarrama. These data will help both state and non-governmental entities develop appropriate policies and strategies to control the disease. 

## 2. Materials and Methods

### 2.1. Study Area

Saudi Arabia is divided into five regions: northern, southern, central, eastern, and western. The focus of this study was Makkah County, located in the western region of the KSA. Makkah is one of the most frequently visited cities in the world. In addition to tourists and migrant workers, Makkah attracts Muslims worldwide due to its central role in the Umrah and Hajj pilgrimages.

Makkah has a semi-desert climate, but the city experiences irregular rainfall between November and January. It is quite warm in the winter, and the weather, in general, is hot and dry. In general, the soil is rocky, with small hills and mountains spread over the city. Makkah is located at an elevation of 277 m above sea level and 21°25′24′′ north (N) and 39°49′2′′ east (E). The population is 1,675,368, and, with millions of Muslims visiting each year, it is one of the most cosmopolitan cities in the world. The survey area was an urbanized residential area in Makkah. It consists of terraced brick double-story houses and multi-story buildings located in an area of approximately 1200 km^2^. The meteorological factors in the survey area are shown in Table 1.

### 2.2. Methods

The survey area was divided into 14 sections based on local administrative precincts. 

A total of 70 workers (14 team leaders and 56 survey personnel) were selected out of a pool of 600 vector control workers in Makkah. These workers were divided into 14 teams serving each of the administrative areas. All teams were comprehensively trained during a 3-day workshop on theories and practical aspects of the survey method to standardize the procedure to identify the aquatic stages of *Aedes* and sampling of larval breeding sites. WHO guidelines were used as a model and reference for the fieldwork in terms of collection, transportation, and storage [17]. After the Workshop, all participants underwent a practical exam and field demonstration to ensure their ability to accurately and effectively conduct the survey.

Ethical review and approval were granted by the Institutional Scientific Review Board of Faculty of Public Health and Health Informatics, Umm-Al-Qura University, Makkah, Kingdom of Saudi Arabia (Centre of Medicine and Medical Science Research) (project #43409019).

### 2.3. Data Collection

Longitudinal surveys of potential *Aedes* larval breeding sites in the 14 sections were conducted during working weekdays (5 days per week) from January 2015 to December 2015. Each section was surveyed by one team equipped with a car and all necessary equipment to collect and inspect artificial/natural water sources for mosquitoes in aquatic stages. All accessible potential larval breeding sites were carefully inspected for the aquatic stages of *Aedes*, using WHO field inspection kits (plastic cups, pipettes, or ladles), which were adapted and used according to the sizes and types of breeding sites. Larvae were sampled using a dipper or pipettes, where applicable. The samples were immediately placed into labeled (location, sample number, and date of sample) WHO standard plastic vials filled with water. Following this, the vials were placed in a humidified cooler and transported to an entomological laboratory for further identification.

Lastly, larvae were carefully classified according to species with the assistance of dissection and microscopes aided by the Pictorial key for identifying mosquito by Leopoldo M. Rueda [18] and the WHO and other guidelines [19,20,21,22]. A standard format was used to record the data.

For further confirmation, larvae and pupae were reared following the Food and Agriculture Organization of the United Nations (FAO) and the International Atomic Energy Agency (IAEA) “Guidelines for routine colony maintenance of *Aedes* mosquito species” [23,24]. 

During the survey, a total of 18,307 larvae and 1226 pupae were collected. Larval and pupal mortality during the rearing was very low and within the average natural mortality. We planned to exclude any tray with a mortality of 5% or more. However, no tray was excluded as we had no mortality approaching that level. The number of larvae and pupae were counted, and the larval stages identified daily, including the number of larvae and pupae that were dead. Overall, 459 larvae (2.5%) and 12 (1%) pupae died. Emerged adult mosquitoes were measured daily, and their sexes determined.

### 2.4. Data Analysis

Data were collated, tabulated, and cleaned using Microsoft^®^ Excel^®^ for Microsoft 365 MSO (16.0.14026.20270) 64-bit (Microsoft Corporation, Redmond, WA, USA) [25]. Given that the meteorological variables (temperature, humidity, rainfall, etc.) are not normally distributed, Kendall’s tau-b correlation was used to test the association between larval habitat and environmental factors. The correlation was completed using IBM SPSS Statistics (IBM, Chicago, IL, USA) [26]. The level of statistical significance for all analyses was set at *p* < 0.05 using 2-tailed comparisons.

## 3. Results

### 3.1. Prevalence of Aedes aegypti Mosquitoes 

**Figure 1:**
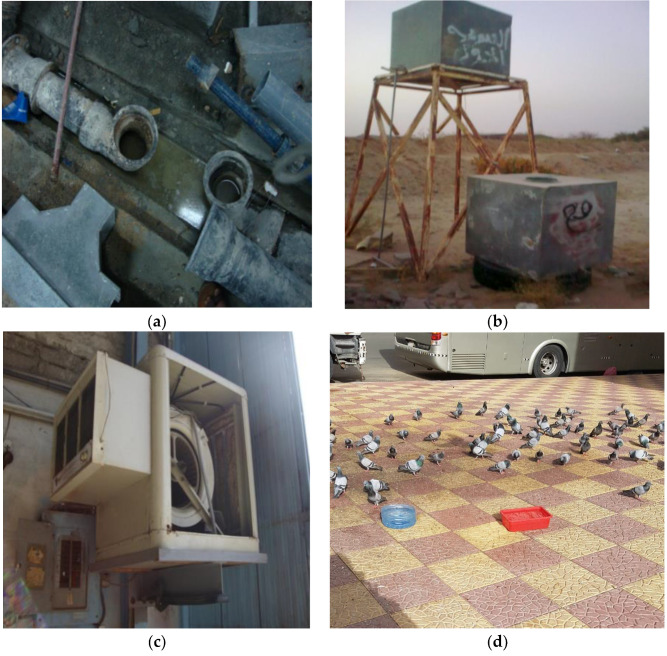
Field photos and examples of potential breeding sites for *Aedes aegypti*. (**a**) Construction site, (**b**) uncovered water tanks, (**c**) air conditioner, and (**d**) bird drinking containers.

**Figure 2:**
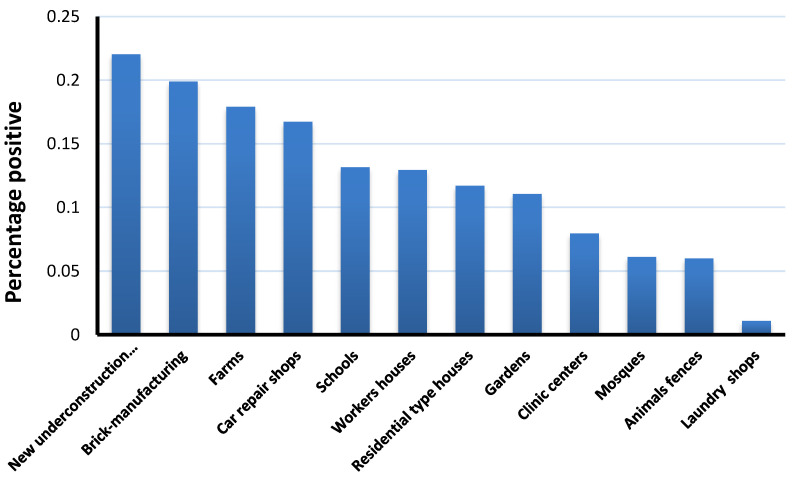
*Aedes aegypti* larval breeding sites.

**Figure 3:**
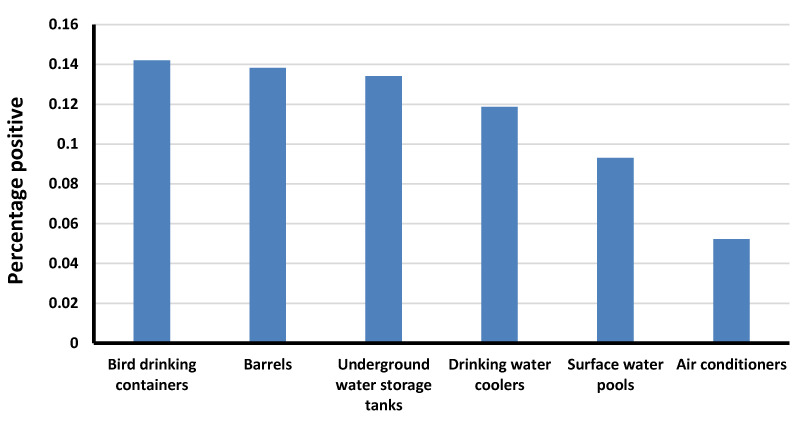
Water sources positive for *Aedes aegypti* larvae.

A total of 42,981 potential *Aedes* larval breeding sites were surveyed (Figure 1). A total of 5403 (12.6%) of these sites had at least one water source for *Aedes aegypti* larvae (Figure 2). Among 15,133 water sources surveyed within these sites, 1815 (12.0%) were positive for *Aedes aegypti* (Figure 3). *Aedes aegypti* was the only *Aedes* species identified during this survey.

### 3.2. Larval Habitats 

There were a variety of larval habitats identified. The most common were new construction sites (both residential and commercial; 22.0%); brick-manufacturing sites (19.9%), and farms (17.9%) (Figure 2). The most common water sources that were positive for *Aedes aegypti* larvae were bird drinking containers (14.2%), barrels (13.8%), and underground water storage tanks at residential and commercial buildings (13.4%) (Figure 3).

### 3.3. Extent of the Mosquito Population

The house index (HI), container index (CI), and Breteau index (BI), commonly used in larval surveys, were used to determine the relative abundance of the mosquito populations. As shown in Table 2, these indices showed high-level infestations of artificial water containers. 

### 3.4. Seasonal Variation and Fluctuation Over Time

**Figure 4:**
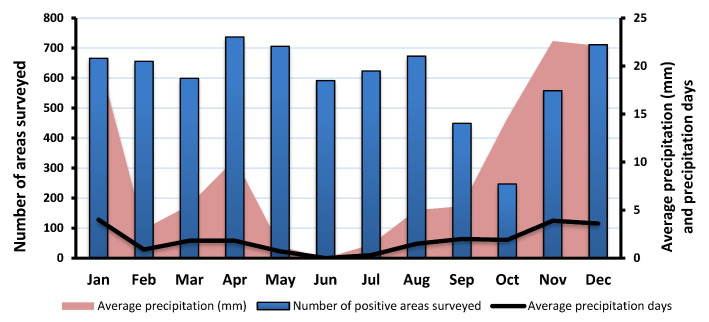
Monthly number of areas surveyed that were positive for *Aedes aegypti* larvae, mean precipitation, and number of days precipitation occurred.

The number of residences positive for *Aedes aegypti* larvae exhibited seasonal variation and fluctuation over time. The number of positive residences remained static, with a minor change from January to March and increasing between April and August. The number of positive residences then decreased substantially in September (*n* = 281) and October (*n* = 124) before rising again from November (*n* = 273), December (*n* = 403), and onwards (Figure 4).

### 3.5. Association between Environmental Factors and Larval Habitat

The correlation between larval habitat and environmental factors (temperature, humidity, and rainfall) mostly showed weak associations between environmental factors and larval habitat (data not shown).

## 4. Discussion

This was a comprehensive study that documented the larval habitats of *Aedes aegypti* in Makkah, Saudi Arabia. Previous studies from Makkah have reported specific aspects of mosquito habitat, surveillance data [27,28], or clinical features of DF [29].

### 4.1. The Role of Domestic Water Storage

Makkah is located in an arid region and has scarce natural water resources. Residents of the city are used to frequent water shortages and tend to store water in the home for extended periods. This practice has contributed to domestic water storage in various containers such as barrels, tanks, and cement tanks. These containers are usually uncovered and placed in shaded areas within courtyards. It is typical for water storage containers also to be constructed inside houses.

Additionally, vast subterranean cement structures are often used for water storage. Networks of internal pipes distribute the water from the reservoir to all parts of the building from a tank on top of the roof. These artificial storage containers have increased the indoor and peridomestic maturation of *Aedes aegypti* in domestic water sources. This supports the earlier observations made by Vezzani [30] and Al-Ghamdi [31].

### 4.2. The Role of Climatic and Meteorological Factors

The climate in Makkah is a desert climate. There is minimal rainfall all year long. The driest months are June and July. Most of the precipitation falls from November to January. A higher number of breeding sites positive for *Aedes aegypti* larvae were detected in July and August, with a decrease from September to April. The highest number of larval breeding sites occurred during the warmest months, indicating that *Aedes aegypti* can survive and reproduce in hot climates. As demonstrated in Figure 4, the lowest number of positive larval breeding sites was detected in October. These results are contrary to earlier research conducted by Aziz et al. (2012). In their survey of household containers, Aziz et al. [28] found that the peak in positive detections was during the wet season. The difference in these findings may result from inconsistent rainfall, with heavier rain during the period covered by the earlier survey. In this survey, the peak periods of larval detection were not a result of the wet season. Instead, the period from August to April was the main period of *Aedes aegypti* larval infestations. Vector control programs should focus on this period to strengthen surveillance systems and develop intervention strategies.

### 4.3. High-Density Breeding Sites

The highest proportion of larval breeding sites were found in new construction sites and brick manufacturing sites. Bird drinking containers, barrels, and underground water storage tanks located underneath residential and commercial building were the most common infested areas surveyed. These findings support those of Aziz et al. [28] and El-Gilany et al. [32]. Therefore, vector control programs should focus on these breeding sites using an integrated approach that targets larval mosquitoes. Such an approach would require environmental management, law enforcement, regulation, public awareness campaigns, health education, personal protection, and dry days. Further research should investigate the methods used by the Cuban and Singaporean governments, as the only countries thus far to have successfully controlled *Aedes aegypti*. In both countries, a combination top-down/bottom-up approach was used.

### 4.4. Extent of the Mosquito Population

The house index (HI), container index (CI), and Breteau index (BI), commonly used in larval surveys, showed a high-level infestation of artificial water containers by mosquito larvae. According to the Pan American Health Organization and the WHO, an area is at a high risk of arbovirus transmission when these indices are above a threshold of 5% for the HI and BI and 3% for the CI. 

### 4.5. Natural and Anthropogenic Breeding Sites

The number of natural water sources that were identified as potential larval breeding sites was small. The identification of these sites was limited to the rainy season, during which there were only two to three rain events. The most significant larval breeding sites were anthropogenic, as demonstrated in Figure 1, Figure 2 and Figure 3. These sites were partially shaded (barrels), completely shaded (subterranean), or wholly exposed (bird drinking water containers) during the entire season, and they included cement structures, barrels, pools, air coolers, animal fences, and animal drinking water containers.

### 4.6. Types of Aedes Identified

Analysis showed that all *Aedes* mosquitoes identified in the survey were *Aedes aegypti* mosquitoes. This finding contradicts the research by Godsy et al. [33], which reported additional species of *Aedes* mosquitoes such as *Aedes *unilineatus**. These findings support that *Aedes aegypti* mosquitoes are capable of continual adaptation to environmental change. Eggs of *Aedes aegypti* can withstand desiccation and survive without water for several months on the inner walls of containers. Vector control programs have not adequately addressed these challenges. These programs struggle to control or eliminate *Aedes aegypti* mosquitoes because of their complex behavior and continuous adaptation to the environment [34].

### 4.7. Seasonal and Demographic Influences

The demographics and geography of Makkah create a real risk of a seasonal increase in the prevalence of DF. More than 10 million people visit the Grand Mosque in Makkah every year, and approximately three million attend the annual *Hajj*, a journey all able Muslims are obliged to make. The seasonal increase in population necessitates an increase in water storage and an increase in potential larval habitats. The influx of pilgrims presents other risks. These travelers, along with many residents of the town, come from a range of backgrounds. Many immigrants and tourists originate from countries where DF is endemic, such as parts of Southeast Asia. These factors combine to increase potential sources of infection. The creation of more favorable potential breeding sites for *Aedes* mosquitoes will increase the prevalence of these mosquitoes. As a result, there is a considerable risk of an outbreak of DF in the near future unless suitable control measures are implemented. If such an outbreak were to occur during the busiest times of the year, it could quickly reach epidemic proportions.

## 5. Conclusions

One of the most efficient methods for controlling *Aedes aegypti* populations relies on eliminating the peridomestic larval breeding sites [34]. The results of this survey suggest that potential larval breeding sites of *Aedes aegypti* will increase, which will create additional challenges. There is a significant risk of epidemics, especially in places where artificial water sources are dominant.

An initial step in controlling *Aedes aegypti* populations would be the targeted elimination of larval breeding sites in high-risk areas. For example, tourists from regions where DF is endemic usually stay around the Masjid al-Haram area. It is recommended that an elimination area free from *Aedes aegypti* be established for a radius of 2 km around the Masjid al-Haram. This can be achieved in many ways, such as strengthening surveillance systems and increasing public awareness, health education about the transmission of DF, drainage of water, improving the design of containers and coverage, a public awareness campaign surrounding dry days for storage containers, the enforcement of regulations, the promotion of physical barriers for containers, and active community participation. In addition, the expansion of *Aedes aegypti* from existing infested areas to nearby new areas of Makkah is highly unpredictable; thus, a consolidated surveillance system for diseases and their vectors is urgently needed. Likewise, further investigation is required into the adaptation of *Aedes aegypti* to arid climate conditions.

Furthermore, new mosquito biocontrol tools that reduce DF transmission risk should be considered an alternative and probably more effective tool than source reduction. Computational models [35,36] and field studies [37,38] have shown that introducing *Wolbachia* strains into natural populations will reduce pathogen transmission and reduce the overall disease burden. Population replacement using a wMel *Wolbachia*-infected *Aedes aegypti* [34] and population removal using sterile *Aedes aegypti* infected with *Wolbachia*-induced cytoplasmic incompatible males [38] have been shown to be successful biocontrol methods.

## 6. Limitations of Study

The issue of the properties of water in the containers surveyed was beyond the scope of this study.

## Figures and Tables

**Table 1:** Meteorological factors in the survey area.

Month	Jan	Feb	Mar	Apr	May	Jun	Jul	Aug	Sep	Oct	Nov	Dec
Record high temperature (°C)	37.4	38.3	42.4	44.7	49.4	49.6	49.8	49.7	49.4	47.0	41.2	38.4
Average high temperature (°C)	30.5	31.7	34.9	38.7	42.0	43.8	43.0	42.8	42.8	40.1	35.2	32.0
Daily mean temperature (°C)	24.0	24.7	27.3	31.0	34.3	35.8	35.9	35.7	35.0	32.2	28.4	25.6
Record low temperature (°C)	11.0	10.0	13.0	15.6	20.3	22.0	23.4	23.4	22.0	18.0	16.4	12.4
Average precipitation (mm)	20.8	3.0	5.5	10.3	1.2	0	1.4	5.0	5.4	14.5	22.6	22.1
Average precipitation days	4.0	0.9	1.8	1.8	0.7	0	0.3	1.5	2.0	1.9	3.9	3.6
Average relative humidity (%)	58.0	54.0	48.0	43.0	36.0	33.0	34.0	39.0	45.0	50.0	58.0	59.0
Mean monthly sunshine hours	260.4	245.8	282.1	282.0	303.8	321.0	313.1	297.6	282	300.7	264.0	248.0
Mean daily sunshine hours	8.4	8.7	9.1	9.4	9.8	10.7	10.1	9.6	9.4	9.7	8.8	8.0

**Table 2:** The density of *Aedes aegypti* infested in potential larval breeding sites.

Months	House Index (HI) Data			Breteau Index (BI) Data			Container Index (CI) Data		
	Number of Inspected Houses	Number of Infested Houses	House Index (HI)	Number of Inspected Houses	Number of Infested Containers	Breteau Index (BI)	Number of Inspected Houses	Number of Infested Containers	Container Index (CI)
January	2368	382	16.1%	2368	438	18.5%	3045	438	14.4%
February	2407	395	16.4%	2407	481	20.0%	3229	481	14.9%
March	2810	317	11.3%	2810	436	15.5%	3712	436	11.7%
April	2082	326	15.7%	2082	467	22.4%	2972	467	15.7%
May	2750	316	11.5%	2750	456	16.6%	3849	456	11.8%
June	2064	141	6.8%	2064	292	14.1%	2951	292	9.9%
July	1874	58	3.1%	1874	253	13.5%	3075	253	8.2%
August	1755	94	5.4%	1755	297	16.9%	2907	297	10.2%
September	2071	189	9.1%	2071	257	12.4%	2697	257	9.5%
October	2205	141	6.4%	2205	156	7.1%	2603	156	6.0%
November	2020	329	16.3%	2020	380	18.8%	2599	380	14.6%
December	2046	362	17.7%	2046	465	22.7%	2760	465	16.8%
Total	26,452	3050	11.5%	26,452	4378	16.6%	36,399	4378	12.0%

## Data Availability

The data presented in this study are available upon reasonable request from the corresponding author and the data custodian, Faculty of Public Health and Health Informatics, Umm-Al-Qura University, Makkah, Kingdom of Saudi Arabia.
